# Immunological Demyelination Triggers Macrophage/Microglial Cells Activation without Inducing Astrogliosis

**DOI:** 10.1155/2013/812456

**Published:** 2013-11-11

**Authors:** Frank Cloutier, Ilse Sears-Kraxberger, Krista Keachie, Hans S. Keirstead

**Affiliations:** ^1^Reeve-Irvine Research Center, Sue and Bill Gross Stem Cell Research Center, School of Medicine, University of California at Irvine, 845 Health Sciences Road, Irvine, CA 92697, USA; ^2^Département de Biologie de l'Université de Moncton, 18 Antonine-Maillet, Moncton, NB, Canada E1A 3E9

## Abstract

The glial scar formed by reactive astrocytes and axon growth inhibitors associated with myelin play important roles in the failure of axonal regeneration following central nervous system (CNS) injury. Our laboratory has previously demonstrated that immunological demyelination of the CNS facilitates regeneration of severed axons following spinal cord injury. In the present study, we evaluate whether immunological demyelination is accompanied with astrogliosis. We compared the astrogliosis and macrophage/microglial cell responses 7 days after either immunological demyelination or a stab injury to the dorsal funiculus. Both lesions induced a strong activated macrophage/microglial cells response which was significantly higher within regions of immunological demyelination. However, immunological demyelination regions were not accompanied by astrogliosis compared to stab injury that induced astrogliosis which extended several millimeters above and below the lesions, evidenced by astroglial hypertrophy, formation of a glial scar, and upregulation of intermediate filaments glial fibrillary acidic protein (GFAP). Moreover, a stab or a hemisection lesion directly within immunological demyelination regions did not induced astrogliosis within the immunological demyelination region. These results suggest that immunological demyelination creates a unique environment in which astrocytes do not form a glial scar and provides a unique model to understand the putative interaction between astrocytes and activated macrophage/microglial cells.

## 1. Introduction 

Myelin represents a nonpermissive substrate for neuronal adhesion, sprouting, and neurite growth [1, 2], and several myelin-associated inhibitor proteins have been identified including the myelin-associated glycoprotein (MAG) [3, 4], oligodendrocyte myelin glycoprotein (OMgp) [5, 6] and Nogo-A [7–9]. Since then, numerous studies have been dedicated to understand the mechanisms underlying the action of these inhibitory molecules [10–12]. Previous studies in our laboratory and others have used immunological demyelination to address myelin-associated inhibition and provide a permissive environment for axonal regeneration. Immunological demyelination involves the intraspinal injection of antibodies to galactocerebroside (GalC), the major sphingolipid in myelin, plus complement proteins, and results in a well-defined region of complete demyelination that spares oligodendrocytes. This treatment paradigm has been shown to promote axonal regeneration following spinal cord injury in embryonic chicks [13], hatchling chicks [14], and adult rats [15–18]. 

One major impediment for axon regeneration following CNS injury is the formation of a glial scar [19, 20]. This response is preceded by the transition of resident astrocytes into a reactive state rapidly following injury. Reactive astrocytes are characterized by a cellular hypertrophy and dramatic changes in gene regulation [21–24]. Notably the upregulation of GFAP has been widely used as marker of astrogliosis.

The role of reactive astrocytes in demyelinating diseases is not fully understood and both protective as well as deleterious effects are being discussed [25–27]. The presence of astrogliosis has been suggested to contribute with the failure of remyelination in many demyelinating pathologies and experimental models of demyelination [28–33]. In our model, remyelination begins 10 to 14 days following intraspinal injection of antibodies to GalC and remyelination of all axons is evident by 4 weeks [17]. Thus, the ability of immunological demyelination regions to sustain axonal regeneration and remyelination suggests that astrogliosis is not induced by the catastrophic destruction of myelin in this model. Such a possibility would seem paradoxical, given that astrogliosis is a ubiquitous response to different insults to the adult CNS including trauma, toxic lesion, genetic, and degenerative diseases [21, 24, 34].

 In the present study we first compared the astrogliosis and macrophage/microglial cells responses 7 days after either immunological demyelination or a stab injury to the dorsal funiculus. Secondly, we compared the astrogliosis response following a stab or hemisection injury to the spinal cord dorsal funiculus within regions of immunological demyelination. Our data shows that immunological demyelination induced a robust macrophage/microglial cells activation which is not accompanied by astrogliosis either when induced alone or followed by an injury.

## 2. Materials and Methods

 Adult female Sprague Dawley rats (200-220 g, 6–8 weeks old; *n* = 40) were anaesthetized with an intraperitoneal injection of 7.5 mg/kg Rompun (Phoenix Pharmaceutical Inc., St. Joseph, MO) and 60 mg/kg Xylazine (Phoenix Pharmaceutical Inc., St. Joseph, MO). All procedures were approved by the Institutional Animal Care and Use Committee of the University of California at Irvine. 

### 2.1. Experimental Groups

 To determine whether immunological demyelination alone induced astrogliosis and activation of macrophage/microglial cells, 10 animals received an injection of GalC antibodies plus serum complement proteins into the dorsal column and were killed 7 days later for immunohistochemical (*n* = 6) or immunoelectron microscopic (*n* = 4) analyses of astroglial reactivity. To determine the extent of astrogliosis following injury, 6 animals received a stab injury in the dorsal column and 6 animals received laminectomy only and were killed 7 days later. To determine whether astrogliosis will be induced within regions of immunological demyelination accompanied with a disruption of the blood brain barrier, 12 animals received an injection of GalC antibodies plus serum complement proteins into the dorsal column, followed by a spinal cord stab wound injury to the dorsal column (*n* = 6) or dorsal spinal cord hemisection injury (*n* = 6) 24 hours later, and were killed after further 7 days. 6 uninjured, normally myelinated animals served as a control group. 

### 2.2. Immunological Demyelination

The dorsal region between the neck and hindlimbs, extending approximately 2 cm bilaterally from the spine, was shaved and disinfected with serial Providone iodine scrub and 70% ethanol scrubs. A midline incision exposed the spinal column at the level of T8–T11 and the paravertebral muscles were dissected bilaterally to visualize the transverse processes. Laminectomy was performed at T10. After stabilization of the spinal column by clamping the T9 and T11 vertebrae, direct injection into the exposed dorsal funiculus was performed using a silicon-coated glass micropipette (with outer diameter of 50–80 *μ*m) attached to a 10 *μ*L Hamilton syringe (Hamilton, Reno, NV) mounted on a micromanipulator. Injections consisted of polyclonal anti-GalC antibody (Chemicon International Inc., Temecula, CA) at a dilution of 1 : 2 with 33% guinea pig complement (ICN, Aurora, OH) in artificial cerebrospinal fluid (aCSF, Harvard apparatus, Holliston, MA). Rats received a total volume of 4 *μ*L injected over 3 minutes. Muscle layers were then sutured and the superficial tissue and skin were closed with wound-clips. 

### 2.3. Spinal Cord Injury

 In animals that received immunological demyelination, the laminectomy site was reexposed 24 hours after intraspinal injection. In animals that had not previously received a demyelinating injection, laminectomy was performed as outlined above. For stab injury, a sterile 25 gauge stainless steel needle (outer diameter of 0, 51 mm) was inserted immediately next to the dorsal vein and lowered 1.2 mm into the dorsal funiculus. For hemisection injury, a microlesion knife (Fisher Scientific, Pittsburgh, PA) marked at 1.2 mm from the tip was lowered into the spinal cord at a depth of 1.2 mm from the dorsal-most point and drawn laterally across the dorsal aspect of the spinal cord. This procedure was then repeated in the opposite direction. Immediately following surgery animals received subcutaneous saline and were maintained on an isothermic pad until alert and mobile. Animals were inspected for weight loss, dehydration, and distress, with appropriate veterinary care as needed.

### 2.4. Histochemical Staining

Animals were sacrificed 7 days following the injection of the demyelinating agent, or 7 days following injury. Animals were terminally anaesthetized and transcardially perfused with 50 mL of 0.1 M phosphate buffered saline (PBS) containing 28 IU/mL heparin (Acros, New Jersey, NY) followed by 250 mL of 4% paraformaldehyde (Fisher Scientific, Pittsburgh, PA) in 0.1 M phosphate buffer (PB) pH 7.4. Spinal cord regions extending 1 cm above and 1 cm below the injection/injury site were postfixed in 4% paraformaldehyde in 0.1 M PB overnight then transferred to 25% sucrose (Fisher Scientific, Pittsburgh, PA) for 24 hours. Spinal cords from stab-injured animals were cut into 1 mm transverse blocks and processed so as to preserve the rostral caudal sequence and orientation. Spinal cords from hemisection-injured animals were cut in the longitudinal plane. 20 *μ*m transverse or longitudinal sections were cut on a cryostat and mounted on gelatin-coated slides (Fisher Scientific, Pittsburgh, PA). 

Rabbit anti-cow glial fibrillary acidic protein (GFAP) antibodies (Dako, Carpinteria, CA) were used at a dilution of 1 : 2000 in 0.5% normal goat serum (NGS) in PBS (Chemicon International Inc., Temecula, CA). Mouse anti-rat CD68 (ED-1 clone) antibodies (Serotec, Oxford, UK) were used at a dilution of 1 : 200 in 0.5% NGS in PBS. Primary antibodies were incubated overnight at 4°C. Goat anti-rabbit or goat anti-mouse secondary antibodies (Alexa Fluor 488 or 594, Molecular Probes, Eugene, OR) were used at a dilution of 1 : 200 in 10% NGS in PBS. Rabbit anti-goat secondary antibodies (Alexa Fluor 546, Molecular Probes, Eugene, OR) were used at a dilution of 1 : 400 in 10% rabbit serum in PBS. After 2 hours of incubation, all sections were washed in PBS and incubated for 5 minutes at room temperature in Hoechst solution (1 : 5000 in PBS; Molecular Probes, Eugene, OR). Standard immunohistochemical controls were included in each run. Sections were viewed and digitally photographed using an Olympus AX-80 microscope using OLYMPUS MicroSuite B3SV software (Olympus America Inc., Melville, NY).

Dorsal columns and demyelinated regions were delineated using cryostat-sectioned tissue stained with eriochrome cyanine R for myelin. Serial sections to those used for immunohistochemical staining were incubated at room temperature with eriochrome cyanine R (Sigma, St. Louis, MO) for 10 minutes, rinsed, and then differentiated with a 10% ferrous oxide solution (Sigma, St. Louis, MO) for 5 minutes. Morphometric analysis of dorsal column and demyelinated regions using eriochrome cyanine R-stained sections was accomplished by tracing dorsal columns and demyelinated regions within digitally photographed fields using the OLYMPUS MicroSuite B3SV software (Olympus America Inc., Melville, NY).

### 2.5. Electron Microscopy

Animals were transcardially perfused with 50 mL of 0.1 M PBS containing 28 IU/mL heparin (Acros Inc., Geel, Belgium) followed by 250 mL of 4% glutaraldehyde (Fisher Scientific, Pittsburgh, PA) in 0.1 M phosphate buffer pH 7.4. Spinal cord regions extending 1 cm above and 1 cm below the injection/injury site were extracted and postfixed for 24 hours in 4% glutaraldehyde. Spinal cords were cut into 1 mm transverse blocks and processed so as to preserve their craniocaudal sequence and orientation. The tissue blocks were rinsed in 0.1 M PB pH 7.4 for 30 min, then exposed to 1% OsO_4_ (Electron Microscopy Sciences, Fort Washington, PA), dehydrated in ascending alcohols, soaked in propylene oxide (Electron Microscopy Sciences, Fort Washington, PA), and embedded in Spurr resin (Electron Microscopy Sciences, Fort Washington, PA) according to standard protocols [17]. Transverse 1 *μ*m semithin sections were cut from the cranial face of each block, stained with alkaline toluidine blue to located the demyelinated regions (Sigma, St. Louis, MO), cover-slipped, and examined by light microscopy on an Olympus AX-80 microscope using OLYMPUS MicroSuite B3SV software (Olympus America Inc., Melville, NY). 

### 2.6. Immunoelectron Microscopy

 40 *μ*m vibratome sections of glutaraldehyde-perfused tissue (above) were cut and pretreated with 10% dimethyl sulfoxide (Fisher Scientific, Pittsburgh, PA) in PBS to enhance antibody penetration, incubated in 0.1% H_2_O_2_ in PBS for 10 min, followed by incubation in PBS containing 10% NGS. Sections were then incubated overnight at 4°C in rabbit anti-cow GFAP antibodies (Dako, Carpinteria, CA) at a dilution of 1 : 2000 in 0.5% NGS in PBS (Chemicon International Inc., Temecula, CA). After several rinses with PBS the sections were incubated in biotinylated goat anti-rabbit IgG (Vector Laboratories, Burlingame, CA) at a dilution of 1 : 400 for 1 hour. Sections were rinsed with PBS and incubated in avidin-biotin-peroxidase complex (Vector Laboratories, Burlingame, CA) at a dilution of 1 : 100 for 1 hour, washed with PBS and postfixed with 2% glutaraldehyde (Fisher Scientific, Pittsburgh, PA) in PBS, rinsed with PBS and visualized with the chromogen DAB (Vector Laboratories, Burlingame, CA). Sections were postfixed in 1% OsO_4_ (Electron Microscopy Sciences, Fort Washington, PA) for 1 hour, dehydrated, immersed in propylene oxide (Electron Microscopy Sciences, Fort Washington, PA), flat-embedded in Spurr resin (Electron Microscopy Sciences, Fort Washington, PA) between two pieces of Aclar film (Electron Microscopy Sciences, Fort Washington, PA), and polymerized at 60°C for 24 hours. The embedded sections were glued onto cured resin blocks and 1 *μ*m semithin sections of the dorsal column were cut with a Leica ultramicrotome (Leica, Wetzlar, Germany), stained with toluidine blue (Sigma, St. Louis, MO) and collected on glass slides. Ultrathin sections were cut and collected on coated slotted copper grids (Electron Microscopy Sciences, Fort Washington, PA), stained with lead citrate (Sigma, St. Louis, MO) for 10 min, and examined using a Philips CM 10 electron microscope.

### 2.7. GFAP and ED-1 Positive Cell Counts

To quantify activated macrophages we used the macrophage-activation antigen (ED-1), which labels a glycosylated lysosomal cytoplasmic antigen believed to be coupled with lysosomal compartments in actively phagocytic macrophages and reactive microglial cells [35–37]. To quantify astrocyte we used the GFAP marker. Transverse ED-1-stained or transverse GFAP-stained sections were viewed using an Olympus AX-80 microscope (Olympus America Inc., Melville, NY). For the epicenter of injuries, for regions of distal astrogliosis or regions of immunological demyelination in the dorsal columns were located at 200x magnification. Images were digitally captured and a 50 × 50 *μ*m (2500 *μ*m^2^) grid was overlaid on the images using OLYMPUS MicroSuite B3SV software (Olympus America Inc., Melville, NY). Regions sampled accounted for over 10 percent of the total region of pathology for each section. For macrophages and reactive microglial cells, cells that had a clearly stained nucleus, exhibiting a rounded phagocytic morphology, and ED-1+ markers that clearly delineated the perimeter of the cell were counted. For astrocytes, cells that had a clearly stained nucleus and exhibiting GFAP markers were counted. 

### 2.8. Semiquantitative Analysis of Distal Astrogliosis

The amount of distal astrogliosis was measured using a semiquantitative method involving computer-assisted image analysis of GFAP immunostaining (University of Texas Image Tool program V. 3). Transverse GFAP-stained sections were viewed using an Olympus AX-80 microscope (Olympus America Inc., Melville, NY). Slides contained obvious histological artifacts including tears or holes were omitted from analysis. The analysis was performed on spinal cord section 1 mm rostral to either the injury epicenter or the demyelinating agent injection site (Figure 5(a)). The dorsal columns were digitally captured at 200x magnification and a 100 × 100 *μ*m (10,000 *μ*m^2^) grid was overlaid on the images using OLYMPUS MicroSuite B3SV software (Olympus America Inc., Melville, NY). Nine 10,000 *μ*m^2^ squares within the middle of the dorsal column of control animals (*n* = 6), injured animals without immunological demyelination (*n* = 6) and injured animals (stab injury) with immunological demyelination (*n* = 6), were analyzed. Within each 10,000 *μ*m^2^ region, a threshold value was assigned for positive staining by selecting genuine GFAP-positive cellular staining within the region. This method allowed us to identify only the cells and processes that stained intensely enough to fall within the threshold range first selected manually and then the same threshold range was used for all the sections. The ImageTool program then automated pixel quantification. 

### 2.9. Statistical Analysis

Results are expressed as mean ± SEM. The software GraphPad Prism 4.0 (L a Jolla, CA, USA) was used for all statistical comparisons. Comparison between the numbers of activated macrophage/microglial cells was analyzed using unpaired Student's *t* tests. Data from the semiquantitative analysis of GFAP staining and the quantitative analysis of GFAP-positive cells were analyzed with a one-way ANOVA followed by a *post hoc* Dunnett's Multiple Comparison test. Statistical significance was set at *P* < 0.05.

## 3. Results

### 3.1. Comparison of GFAP Immunoreactivity 7 Days following Immunological Demyelination or following a Stab Wound Injury to the Dorsal Column

**Figure 1:**
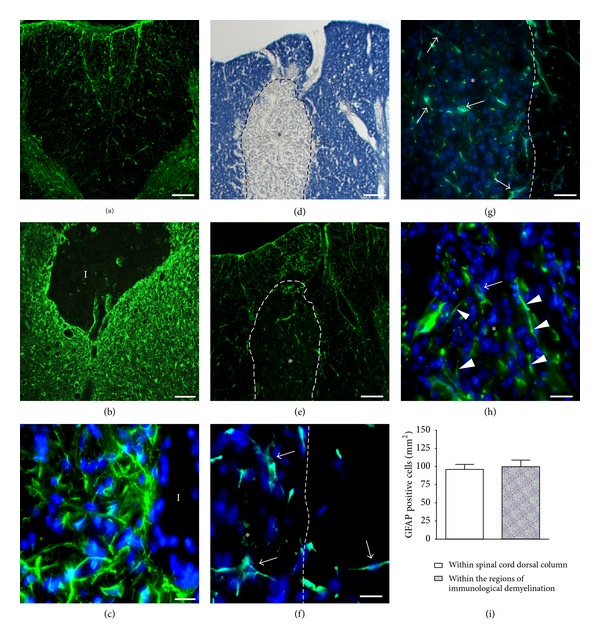
Comparison of the expression of GFAP 7 days after a spinal cord stab injury to the dorsal column or after an injection of GalC antibodies plus complement proteins to the dorsal column. (a) The intensity of GFAP (green) immunoreactivity is low in the uninjured spinal cord dorsal column. (b) A dense ring of reactive astrocytes with intense GFAP (green) expression is evident surrounding the lesion “I.” (c) Higher magnification of the lesion edge: the double staining for GFAP and Hoechst reveals many reactive astrocytes tightly juxtaposed each other. (d) Eriochrome cyanine R staining showing the region of immunological demyelination. (e) GFAP (green) staining of a serial section to (d). (f) Higher magnification view within the region of demyelination showing double staining of GFAP (green) and Hoechst (blue). Note the presence of GFAP positive astrocytes (arrows) close to the edge of immunological demyelination region (white*). (g) GFAP positive astrocytes (arrows) deeper within the region of demyelination. Note the absence of reactive astrocytes at the border zone between normally myelinated and demyelinated area. (h) Higher magnification within the region of demyelination: the double staining for GFAP and Hoechst reveals an astrocyte (arrow) and multiple intermediate GFAP filaments (arrow heads). (i) The numbers of GFAP-positive cells within regions of immunological demyelination were similar compared to those within regions of normal dorsal column. Scale bars: (a) and (b) 100 *μ*m; (d), (e), and (g) 50 *μ*m; (c), (f), and (h) 16.6 *μ*m.

In intact rats, regularly spaced, radially oriented GFAP-positive astrocytes were numerous within the dorsal white matter (Figure 1(a)). Stab injury to the dorsal column induced a classic astrocytic reaction characterized by hypertrophy and upregulation of GFAP (green) expression at 7 days after injury (Figure 1(b)). A high density of reactive astrocytes with intense GFAP expression was present within white and gray matter at the level of the injury (Figure 1(b)); the intensity of GFAP immunoreactivity at the injury site was notably increased as compared to that in the uninjured spinal cord (Figure 1(a)). Higher magnification revealed the intensely GFAP-immunoreactive white matter surrounding lesion-induced cavities bordered by a dense meshwork of reactive astrocytes with thick and long processes, which were intensely labeled with GFAP (green) (Figure 1(c)). Distal reactive astrocytes were also present 2 mm rostral and caudal to the injury epicenter (data not shown).

 Intraspinal injection of GalC antibodies plus serum complement proteins induced a well-defined region of demyelination (*) within the dorsal column (Figure 1(d)). The area of immunological demyelination extended 3-4 mm above and 3-4 mm below the site of injection as previously reported [17, 18] (data not shown). Anti-GFAP immunostained serial sections (Figure 1(e)) indicated that immunological demyelination did not induce widespread reactive astrogliosis either within or adjacent to regions of immunological demyelination. Higher magnification showed that the edge of immunological demyelination area (hatched line) contains small non-hypertrophic astrocytes with short processes (arrows) (Figure 1(c)) which contrasted with the edge of injury bordered by a dense meshwork of reactive astrocytes (Figure 1(f)). Astrocytes were also found deeper within the regions of immunological demyelination (arrow in Figure 1(g)) and higher magnification showed nonhypertrophic astrocyte (arrows) and multiple GFAP positive filaments (arrow heads) (Figure 1(h)). This contrasted with the absence of GFAP positive cells and GFAP positive intermediate filaments within the lesion site (I) produced by the stab injury (data not shown). Quantitative analysis of GFAP-positive cells revealed no difference between the numbers of GFAP-positive cells within the normal white matter in the dorsal column compared to those within regions of immunological demyelination (Figure 1(i)). These finding confirm that astrocytes survived within regions of immunological demyelination, indicating that the lack of reactive gliosis within and surrounding regions of immunological demyelination is not due to death of astrocytes. 

**Figure 2:**
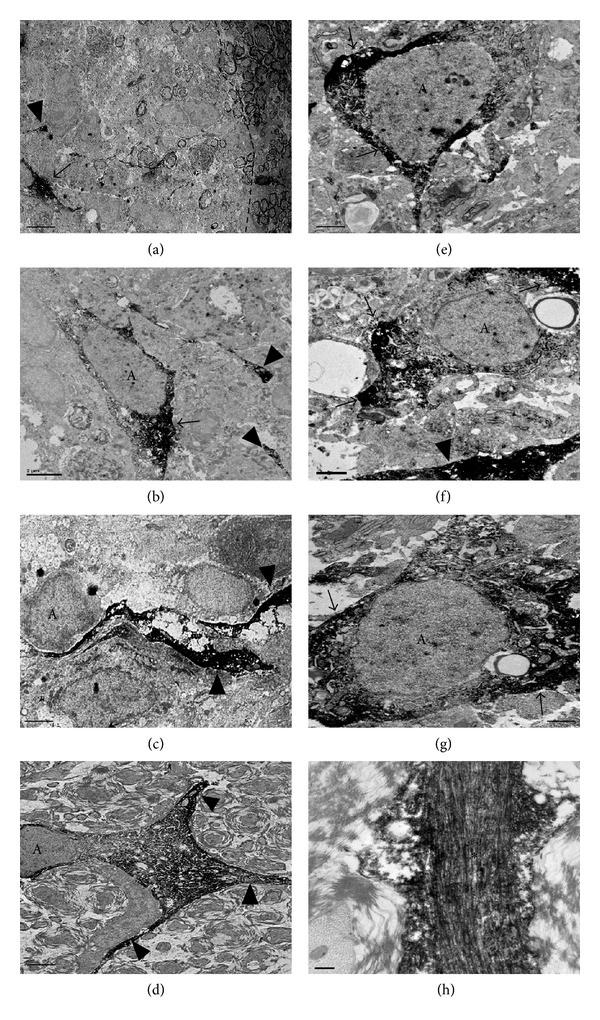
Localization of GFAP-immunoreactivity by immunoelectron microscopy within the region of immunological demyelination 7 days following intraspinal injection of GalC antibodies plus complement proteins. (a) Lower magnification of (b) showing a GFAP-positive astrocyte and the nearby border zone between the normally myelinated and demyelinated region (hatched line). Note the lack of astrogliosis at the border zone. (b)–(g) Astrocyte (A) with GFAP-immunoreactivity (arrows) within the cytoplasmic compartment immediately adjacent to the nucleus and a GFAP-positive process (arrowhead). For comparison, (d) illustrates distal reactive astrocytes 1 mm rostral to the injury epicenter. Note the hypertrophied process and multiple branches (arrowheads). (h) High magnification with focus on GFAP-positive process (black*). Note multiple dark GFAP-positive parallel lines corresponding to intermediate filaments. Scale bars: (a) 5 *μ*m; (b), (c), and (f) 2 *μ*m; (d) 5 *μ*m; (e) and (g) 1 *μ*m; (h) 0,2 *μ*m.

 Further support for the survival of astrocytes and lack of astroglial reactivity within regions of immunological demyelination was obtained from electron microscopic analyses (Figure 2). GFAP immunoreactive cells were present within the regions of immunological demyelination either close to the edge (Figures 2(a)-2(b)) or deeper within the regions of immunological demyelination (Figures 2(c), 2(e), 2(f), and 2(g)). GFAP immunoreactivities were found within the cytoplasmic compartment immediately adjacent to the nucleus (arrows) and within processes (arrowheads). A lower magnification view (Figure 2(a)) from Figure 2(b) indicates a lack of astrogliosis within the region of immunological demyelination or at the edge (hatched line) with myelinated white matter. For comparison, distal reactive astrocyte 1 mm rostral to the injury epicenter without immunological demyelination possesses a hypertrophied cell body, numerous spread processes (Figure 2(d)), and thick bands of intermediate filaments within their processes (Figure 2(h)). 

### 3.2. Macrophage/Microglial Cell Response following Spinal Cord Injury and Immunological Demyelination

**Figure 3:**
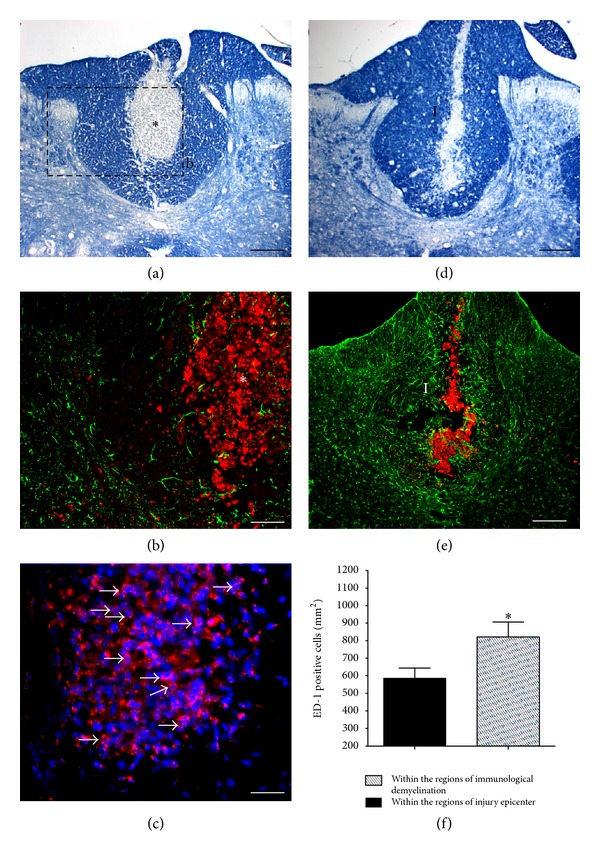
Macrophage-activation antigens (ED-1) 7 days following intraspinal injection of GalC antibodies plus complement proteins or a spinal cord stab injury to the dorsal column. (a) Eriochrome cyanine R-stained transverse section showing the region of immunological demyelination (*). (b) Double stained GFAP (green) and ED-1 (red) serial section to (a). The inset highlights the numerous ED-1 positive cells within the region of immunological demyelination (white*) and the absence of astrogliosis. (c) Localization of ED-1 positive cells (arrows) within the region of immunological demyelination (hatched line). Activated macrophages characterized by their large round cell bodies and their high immunoreactivity for ED-1 are distributed homogeneously throughout the whole region of immunological demyelination. (d) Eriochrome cyanine R-stained transverse section showing the injury site (I). (e) Double stained GFAP (green) and ED-1 (red) serial section to (d). Numerous ED-1 positive cells are present within the injury site bordered by numerous GFAP positive-hypertrophic astrocytes. (f) Quantitative comparisons between the number of ED-1+ cells per mm^2^ found within injury epicenters and within regions of immunological demyelination (*), *P* < 0.05. Scale bars: (a), (d), and (e) 200 *μ*m; (b) and (c) 50 *μ*m.

The presence of activated macrophage/microglial cells within regions of immunological demyelination has been previously reported [17, 38]. Likewise, the response of macrophage/microglial cells following hemisection, crush, or contusion injury of the rat spinal cord has been thoroughly described [39–42] and found to be maximal between 3 and 7 days after injury. In the present study, we quantitatively compared macrophage/microglial cell response 7 days following immunological demyelination or stab injury to the dorsal column. Eriochrome cyanine R staining revealed well-defined regions of immunological demyelination within the center of the dorsal column (Figure 3(a)). Double anti-ED-1 (red) and GFAP (green) immunostained serial sections to A (Figure 3(b)) revealed that immunological demyelination is accompanied by a robust activation of macrophage/microglial cells without astrogliosis response. Numerous activated macrophages characterized by their large round cell bodies and their high immunoreactivity for ED-1 were distributed homogeneously throughout the whole region of immunological demyelination (arrows, Figure 3(c)). Notably, macrophage/microglial cells were restricted almost exclusively to regions of immunological demyelination, with only a few cells outside of the region of immunological demyelination (Figure 3(c)) or rostral and caudal to the region of immunological demyelination (data not shown). Supporting our previous observations in this study, the region of immunological demyelination was accompanied by astrogliosis (Figure 3(b)). 

For the stab injury to the dorsal funiculus, the trace of the needle tract and accompanying damage to the dorsal column were revealed by the eriochrome cyanine R staining (Figure 3(d)). ED-1+ cells were found mainly at the injury epicenter in all cases (Figure 3(e)). Few ED-1 positive cells were scattered throughout peripheral zones of ventral and lateral white matter rostral and caudal to the epicenter (data not shown). Quantitative analysis showed a statistically significant difference between the number of ED-1+ cells per mm^2^ found within the injury epicenters and within regions of immunological demyelination without injury (*) *P* < 0.05 (Figure 3(f)). These findings indicate that both immunological demyelination and stab injury induce a strong macrophage/microglial cell response despite differing astrogliotic responses. 

### 3.3. Combination of Immunological Demyelination and Spinal Cord Injury

 Previous studies by Silver group and others have observed a correlation between the most significant blood brain barrier (BBB) breakdown and the greatest astrogliosis formation [43–46]. To determine whether a breakdown of BBB within the regions of immunological demyelination induced astrogliosis, we first created a region of immunological demyelination in the dorsal column and then 24 h later an hemisection or a stab injury was performed. 

**Figure 4:**
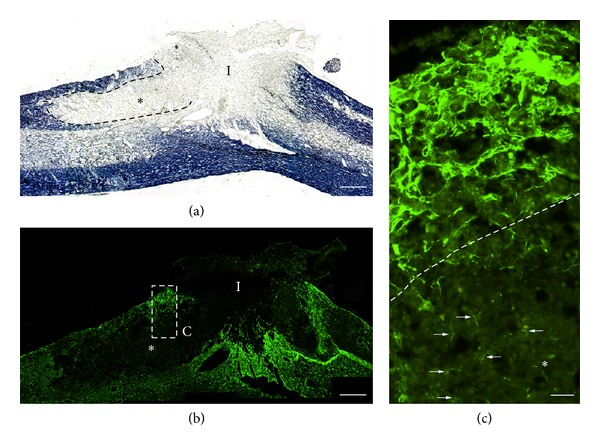
Immunological demyelination prevents astroglial hypertrophy following dorsal hemisection injury. (a) Eriochrome cyanine R-stained parasagittal section showing the extent of the demyelination (*) in the dorsal column rostral to the hemisection injury site (I). (b) GFAP-stained parasagittal serial section to (a). Astroglial hypertrophy is evident surrounding the hemisection injury, but is absent within the region of immunological demyelination (*). (c) Higher magnification of box in (b) showing the edge of the region of gliosis within normal white matter above the region of demyelination (hatched line indicates the border of the region of demyelination). The arrows demark a few of the many astrocytes present within the region of demyelination. Note that these astrocytes are small and have short and thin processes characteristic of quiescent astrocytes, which contrast with the hypertrophic astrocytes within the region of gliosis immediately above. Scale bars: (a) and (b) 400 *μ*m; (c) 50 *μ*m.

Findings from hemisection are presented in Figure 4. In hemisection-injured animals, eriochrome cyanine R-stained midline longitudinal sections clearly revealed the dorsal and ventral white matter tracks (blue stain) within the spinal cord (Figure 4(a)). Regions of immunological demyelination appeared as white bands within the dorsal column and extended several millimeters rostral and caudal to the hemisection injury site (labeled I in Figure 4(a)). Serial sections stained for GFAP revealed robust astroglial hypertrophy and GFAP expression around the injury site but not within regions of immunological demyelination; astrocytes within regions of immunological demyelination did not display the intense GFAP staining typical of reactive astrocytes either at a distance from or immediately adjacent to the base of the lesion (Figure 4(b)). Higher magnification of the white matter adjacent to the injury site confirmed robust astroglial hypertrophy and GFAP expression within normally myelinated white matter, but a lack of astroglial reactivity within regions of immunological demyelination; GFAP immunostaining within regions of immunological demyelination revealed astrocytes with short, thin processes characteristic of quiescent cells (arrows in Figure 4(c)). The number and distribution of astrocytes within regions of immunological demyelination appeared similar to the number and distribution of astrocytes within normal white matter.

**Figure 5:**
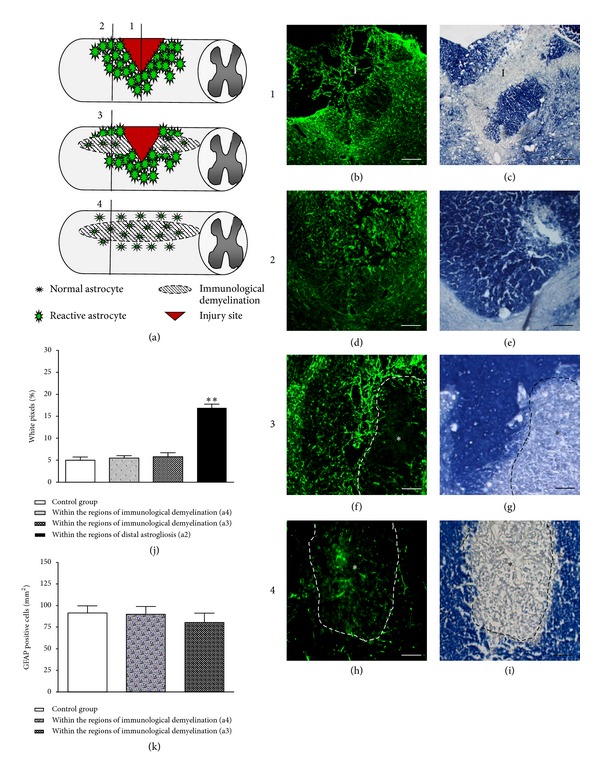
Immunological demyelination attenuates the induction of distal reactive. (a) Schematic drawing of different experimental groups and where analysis of white pixel and GFAP-positive cell counts were conducted. (b) GFAP-stained serial section to (c). The injury site is surrounded by wide spread astrogliosis in the gray and the white matter. (c) Eriochrome cyanine R-stained transverse section showing the injury site (I). (d) GFAP-stained serial section to (e). Astrogliosis is still evident 1 mm rostral the injury epicenter. (f) GFAP-stained serial section to (g). (f) Astrogliosis is markedly reduced within the region of demyelination (*, hatched line). (e) Eriochrome cyanine R-stained transverse section showing the region of demyelination 1 mm rostral the injury epicenter. (j) Whereas there is a significant increase in % of GFAP staining 1 mm rostral to the injury epicenter, regions of immunological demyelination showed a similar percentage of GFAP staining compared to normal white matter. (k) The numbers of GFAP-positive cells within regions of immunological demyelination either alone or 1 mm rostral to the injury epicenter are similar compared to those of the normal dorsal column. **: *P* < 0.01. Scale bars: (b)-(c) 100 *μ*m; (d)–(i) 50 *μ*m.

 Quantitative analysis of GFAP-positive cells and semiquantitative analysis of GFAP staining are depicted in Figure 5. Schematic drawing shows where the analyses were conducted (Figure 5(a)). Since it was difficult to distinguish regions of immunological demyelination at the injury epicenters due to the destruction and distortion of tissues, these analyses were conducted 1 mm rostral to the injury epicenters (stab injury) (Figures 5(b)-5(c)) where astrogliosis was still predominant and clear delineated regions of immunological demyelination with eriochrome cyanine R staining could be identified (Figures 5(d)–5(g)). In animals that received a stab injury without previous immunological demyelination, GFAP stained transverse sections revealed a dense population of distal hypertrophic reactive astrocytes intensely expressing GFAP in the dorsal column 1 mm rostral (Figure 5(d)) to the injury epicenter (Figure 5(b)). However, in animals that underwent immunological demyelination, hypertrophic astrocytes were absent within the demyelinated area (*) but present in surrounding tissue (Figure 5(f)). GFAP pixel quantification revealed a statistically significant decrease within regions of immunological demyelination (hatched line) compared to regions of astrogliosis without immunological demyelination 1 mm rostral to the injury epicenter; 16.74 ± 1.18% to 5.59 ± 0.82%, *n* = 6, *P* < 0.01 (Figure 5(j)). No statistical difference was found between the pixel content within regions of immunological demyelination and within the dorsal column of control animals (Figure 5(j)). 

## 4. Discussion

 In this study we report that immunological demyelination is not accompanied by astrogliosis despite causing intense macrophage/microglial activation and document a novel animal model to understand the putative physiological response of astrocytes during inflammation in the central nervous system.

### 4.1. Survival of Astrocyte within Immunological Demyelination

The lack of astroglial reactivity within regions of immunological demyelination raises the question whether this treatment might just kill astrocytes. For instance, other models of demyelination, such as intraspinal injection of ethidium bromide, create a myelin-free region in which astrocytes die and consequently no astroglial reactivity is seen within the myelin-free region [47–49]. In the present study, immunohistochemical and immunoelectron microscopic assays revealed GFAP positive cells within regions of immunological demyelination, indicating that astrocytes survived. Furthermore, our quantitative analysis indicated that regions of immunological demyelination contained a number of GFAP-positive cells that were not statistically different compared to normal dorsal white matter. Similarly, one previous study using intraspinal injection of anti-GalC observed a survival of astrocytes but a reduction of GFAP staining, although no quantitative data was presented [50]. It is noteworthy that immunological demyelination is followed by complete remyelination by oligodendrocytes [17] and numerous studies showed that astrocytes are needed for oligodendrocyte remyelination [51–55]. Although we showed that immunological demyelination with anti-GalC does not kill astrocytes, further investigation will be needed to clarify whether regions of immunological contains newly formed astrocytes or dying astrocytes. 

### 4.2. Why Immunological Demyelination Is Not Accompanied by Astrogliosis?

Increased expression of GFAP is a hallmark of astrogliosis and our semiquantitative analysis indicated that regions of immunological demyelination contained a GFAP density that was not statistically different from the GFAP density in normal spinal cords. Astrocytes are distributed throughout the whole CNS and become reactive in response to essentially any disturbance, including trauma, stroke, neurotoxic damage, genetic, and neurodegenerative diseases, for reviews [21, 24, 34]. Regarding animal models of demyelination, an increase in astrogliosis has been previously reported during cuprizone-induced demyelination [56–58] as well as with the lysolecithin demyelination model [59–61]. Immunological demyelination itself represents as well an insult to the CNS. Immunological demyelination involves the targeting of antibodies against the major sphingolipid in myelin (galactocerebroside) and the subsequent activation of the complement cascade, leading to the formation of membrane attack complexes and myelin destruction within hours of administration [13, 38, 62]. 

Many different types of molecules that can be generated through a wide variety of different mechanisms are able to trigger features of reactive astrogliosis. Molecular mediators of reactive astrogliosis can be released by any cell type in central nervous system tissue, including neurons, microglia, oligodendrocyte lineage cells, endothelia, leukocytes, and astrocytes, reviewed in [23, 24]. Immunological demyelination is accompanied by a large population of activated macrophage/microglial cells. Activated macrophage/microglial cells can release putative triggers of astrogliosis [22] like IL-1*β* [63–68], IL-6 [69–71] and TNF-*α* [67]. Conversely, the anti-inflammatory cytokine interleukin (IL)-10, a potent inhibitor of cytokine synthesis from microglia and macrophages [72–76], has been reported to reduce glial scaring [77]. Other evidence for a role of inflammatory mononuclear cell cytokines as mediators of astrogliosis is coming from studies on CNS development. Indeed, one hypothesis to explain the lack of astrogliosis during CNS development is the relative immaturity of the immune system in neonates compared to adults [78–81]. Yong and coworkers showed that the inability of neonatal astrocytes to become reactive can be reversed by microinjection of gamma-IFN, IL-1, IL-2, IL-6, TNF-*α*, and macrophage colony stimulating factor (M-CSF), which resulted in a significant increase of astrogliosis in neonatal mouse brain, similar to that seen in an adult [82]. In the cuprizone-induced demyelination model, astrogliosis promptly followed macrophage/microglia activation [57] and an increased expression of many inflammatory cytokines including TNF-*α* and IL-1*β* has been demonstrated within regions of demyelination. Using the same model, Skripuletz and colleagues demonstrated that reactive astrocytes provide the signal environment that forms the bases for the recruitment of microglia to clear myelin debris [58]. With the lysolecithin demyelination model, many cytokines triggers of astrogliosis including TNF-*α* and IL-1*β* are release as well [61, 83, 84]. Therefore the presence of a large population of activated macrophage/microglial cells within regions of immunological demyelination and the concomitant lack of astrogliosis is quite unique. Clearly a different signaling occurs within regions of immunological demyelination which does not encourage astrocytes to become hypertrophic and form a glial scar. 

The lack of blood brain barrier (BBB) breakdown represents another hypothesis. Previous studies by Silver group and others have observed a correlation between the most significant BBB breakdown and the greatest scar formation [43–46]. These observations lead to the hypothesis that extravasation of serum components might trigger astrogliosis. Supporting this view, Schachtrup et al. [85] demonstrated that the blood protein fibrinogen, which leaks into the CNS immediately after BBB disruption or vascular damage, serves as an early signal for the induction of glial scar formation via the TGF-*β*/Smad signaling pathway [85, 86]. It is noteworthy that we observed reactive astrocytes around the needle insertion site for the injection of anti-galactocerebroside antibodies and complement proteins serum suggests that the lack of astrogliosis might correlate with the lack of BBB breakdown. However, this hypothesis does not explain why distal astrocytes do not become reactive with hypertrophied bodies within regions of immunological demyelination overlapping the extended area of distal astrogliosis several millimeters above or below the stab wound injury site. It is likely that distal astrocytes caudal or rostral to the lesion are exposed to all the astrogliosis mediators released following the BBB breakdown and should become reactive and hypertrophied. Again, this observation raises the hypothesis that a different signaling occurs within regions of immunological demyelination compared to other experimental models of demyelination.

In conclusion, our results indicate that immunological demyelination triggers a robust activated macrophage/microglial cells response without inducing astrogliosis. Nonetheless, several questions remain to be addressed. How does immunological demyelination affect astrocyte metabolism and expression of other markers of reactive astrocyte? Has immunological demyelination simply slowed the astrocyte response, killed a percentage of astrocytes, or even metabolically damaged them so as to decrease their ability to generate GFAP? Immunological demyelination offers a unique model to understand the putative physiological response of astrocytes and their molecular interplay with activated macrophage/microglial cells during inflammation in the central nervous system.
